# Confinement-Controlled Water Engenders Unusually High Electrochemical Capacitance

**DOI:** 10.1021/acs.jpclett.3c01498

**Published:** 2023-07-17

**Authors:** Svetlana Melnik, Alexander Ryzhov, Alexei Kiselev, Aleksandra Radenovic, Tanja Weil, Keith J. Stevenson, Vasily G. Artemov

**Affiliations:** ‡ Atmospheric Microphysics Department, Leibniz Institute for Tropospheric Research, 04318 Leipzig, Germany; § Center for Low-Emission Transport, Austrian Institute of Technology, 1210 Vienna, Austria; ∥ Institute of Meteorology and Climate Research, Karlsruhe Institute of Technology, 76021 Karlsruhe, Germany; ⊥ Institute of Bioengineering, École Polytechnique Fédérale de Lausanne (EPFL), CH-1015 Lausanne, Switzerland; # Max Planck Institute for Polymer Research, Ackermannweg 10, 55128 Mainz, Germany; ∇ Skolkovo Institute of Science and Technology, 121205 Moscow, Russia

## Abstract

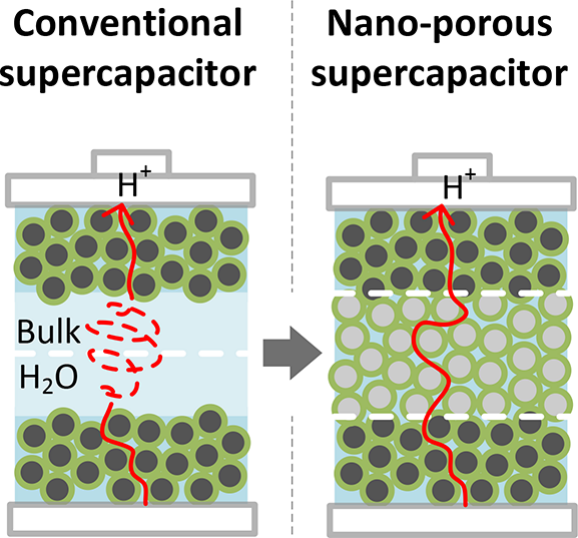

The electrodynamics of nanoconfined water have been shown to change dramatically compared to bulk water, opening room for safe electrochemical systems. We demonstrate a nanofluidic “water-only” battery that exploits anomalously high electrolytic properties of pure water at firm confinement. The device consists of a membrane electrode assembly of carbon-based nanomaterials, forming continuously interconnected water-filled nanochannels between the separator and electrodes. The efficiency of the cell in the 1–100 nm pore size range shows a maximum energy density at 3 nm, challenging the region of the current metal-ion batteries. Our results establish the electrodynamic fundamentals of nanoconfined water and pave the way for low-cost and inherently safe energy storage solutions that are much needed in the renewable energy sector.

Environmentally neutral energy storage for portable and stationary applications is a cornerstone of the current global energy trends,^1^ intensifying the development of sustainable batteries. Aqueous systems for electrochemical energy storage combine inherent environmental neutrality with safety and low cost.^2−4^ However, the insufficient understanding of the dynamic structure of aqueous interfaces under different boundary and electrodynamic conditions^5,6^ limits the optimization of electrodes and separators. The potential of water-based electric energy systems is largely unexploited.^7−9^

Previous studies showed water anomalies near solid–liquid interfaces.^10−25^ The interfacial water possessed a behavior different from that of the bulk water. Most often the altered dielectric properties of water are reported for its contact with carbon-based materials.^12,13,17,26,27^ Electron-conducting forms of carbon (e.g., nanotubes or graphene) have been shown to affect the viscosity of the interfacial water^12,27,28^ and provide high specific electric capacitance of up to 500 F/g.^29,30^ Thus, they represent ideal electrodes for aqueous systems. Dielectric carbon, such as a diamond, has been shown to polarize water and increase the protonic conductivity by 5 orders of magnitude within a nanometer thick interfacial water layer.^18,31^ The anomalous conductivity is close to that of electrolyte solutions and bulk water exhibited at microwave frequencies.^32^ Thus, confined in diamond water can be used as an electrode separator with high proton conductivity. The challenge is to find an optimal membrane electrode assembly (hereafter cell) and pore size distribution to fully exploit the interfacial water properties for energy storage, avoiding bulk water inside and at the contact of the functional layers.

Here, we construct a carbon-based nanoporous electrochemical double-layer capacitor that stores electrical energy without the use of harmful materials. We demonstrate that pure water behaves as an electrolyte under strong confinement, in which intrinsic H_3_O^+^ and OH^–^ ion pairs can be separated and stored more efficiently than in bulk water. We show that the maximum energy density of the water-only battery is achieved at 3 nm pores. Thus, we demonstrate a route to inherently safe electric energy storage and discuss directions for further improvement of the electrochemical performance of water-only batteries.

*Confined-Water Electrochemical Double-Layer Capacitor*. Figure 1A shows a cell assembly that we fabricated to study the charge storage in nanoconfined water. The cell consists of two electrode layers and a dielectric separator (panels D and G of Figure 1). The electrodes are made of fine-grained activated carbon. The separator is made of nanodiamond grains (panels E and H of Figure 1). All layers are pure carbon-based materials (see the Supporting Information). We fabricated several dozen devices with seven selected grain sizes of 5, 18, 40, 80, 120, 200, and 500 nm. This range varies the pore size of the separator of electrodes within 2 orders of magnitude (Figure S1 of the Supporting Information). The grains had a narrow size distribution with a dispersion of less than 40% and were compressed into a porous ceramic with almost the densest packing. Note that, for the randomly shaped densely packed particles, the mean pore size *r* is 3 times smaller than the mean grain size *d* and the net pore volume is 35% of the total volume.^33^

**Figure 1:**
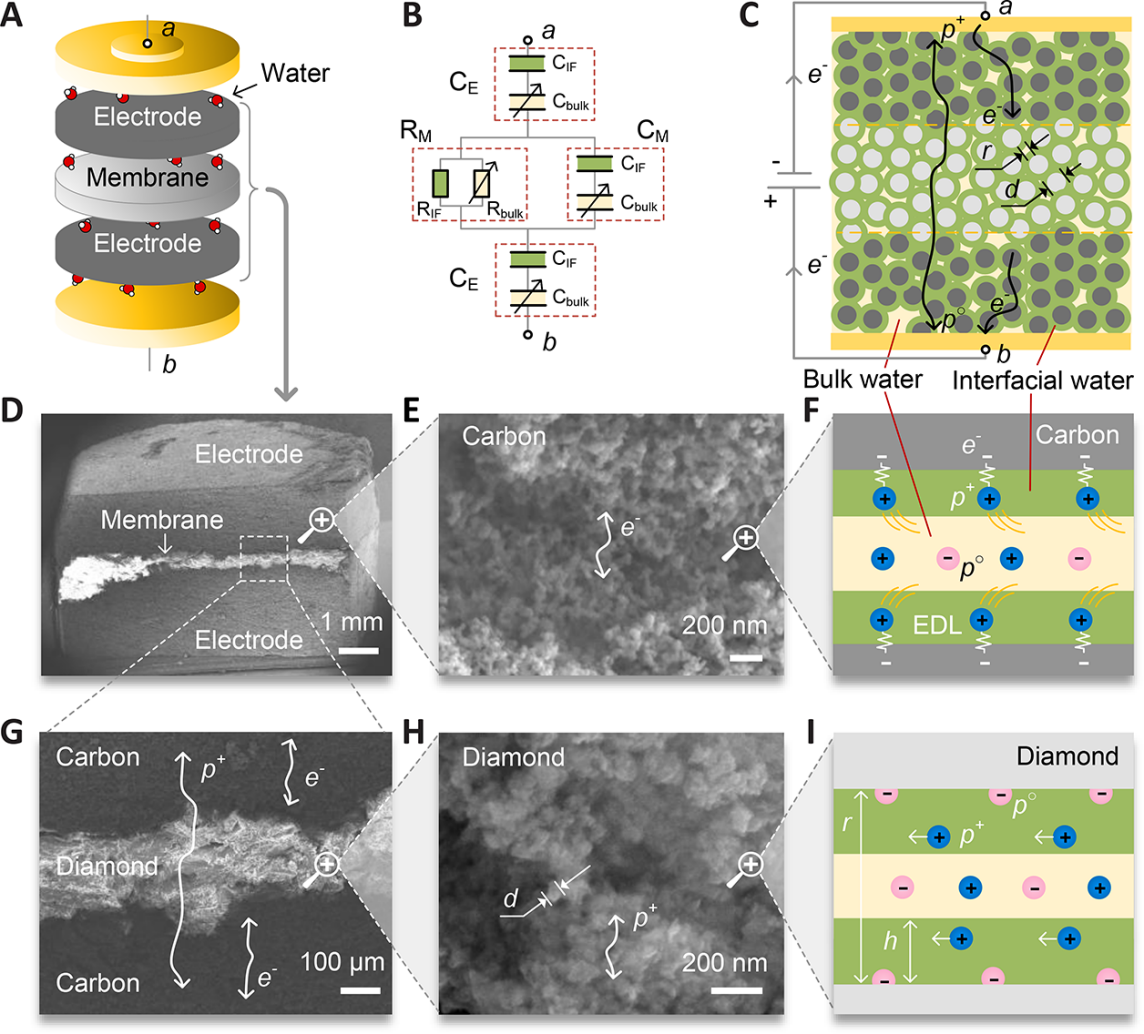
Schematic diagram of the confined-water electric energy accumulator. (A) Layout of the cell. (B) Equivalent electric circuit of the cell. The letters a and b denote the points where the impedance measurements are made. (C) Structural model of the cell. The circles show carbon (dark gray) and diamond (light gray) grains of the diameter *d*. The pore space is filled with water. Interfacial water is shown in green, and bulk water is shown in yellow. Arrows show the route for electronic and protonic conductivity. (D, E, G, and H) Electronic microscopy micrographs of the functional layers at different resolutions (magnifications of 27×, 25000×, 120×, and 125000×, respectively). (F) Model of the interaction of water with the charged carbon surface. (I) Model of the interaction of water with the diamond surface. Plus and minus signs represent excess protons (H_3_O^+^ ions) and proton holes (OH^–^ ions). White arrows with *r* and *h* show the pore size and interfacial water thickness, respectively.

Unlike the previously used carbon-based membrane electrode assemblies,^34,35^ the separator of our cell was made of a nanograined dielectric separator and placed between the electrode layers avoiding gaps with the electrodes. The grains of the electrode and separator formed a continuous percolation network of nanopores without interruption of the interfacial water layers at the layer interfaces (Figure 1C). The pores were filled with water that served as a proton conductor. The equivalent scheme of the cell (Figure 1B) consisted of two capacitors, *C*_E_, and the capacitor–resistor pair, *C*_M_–*R*_M_, representing the electrodes and the membrane separator, respectively. To calculate the parameters at different pore sizes, we considered the confined water to be divided into two parts (Figure 1C): the interfacial water (green) and the bulk water (yellow). Because the interfacial and bulk water have different dielectric properties, each element of the equivalent circuit was represented as a combination of resistances, *R*_bulk_ and *R*_IF_, and capacitances, *C*_bulk_ and *C*_IF_, representing the bulk and interfacial water layers, respectively.

*Electrochemical Performance of the Confined-Water Capacitor*. Figure 2 shows the dependence of the electrodynamic parameters of the cell on grain size *d*. One can see the ionic (protonic) conductivity σ_ab_ (Figure 2A), capacitance *C*_s_ (Figure 2B), charge density *q*, and coulombic efficiency CE = (1 – *j*σ_ab_^–1^*U*_max_^–1^) × 100% ≈ *t*_d_/*t*_c_ × 100%, where *t*_c_ and *t*_d_ are the charge and discharge times, respectively (Figure 2C). All properties change consistently and inversely proportional to *d* for large grain (pore) sizes larger than *d* ≈ 20 nm. For smaller *d*, all of the parameters saturate. The maximum values of the conductivity σ_ab_ and the capacitance *C*_s_, which are related to CE and the charge *q*, are observed for the grain size *d*_max_ ≈ 10 nm or the pore size *r*_max_ ≈ 3 nm (see below). In these pores, the electrical conductivity of pure water is comparable to that of strong electrolytes.^36^ A further decrease of *d* down to the angstrom scale leads to a sharp decrease in all of the cell properties. This happens presumably as a result of the overlapping of the interfacial water layers of the opposite sides of the pore and the subsequent Coulomb blocking of the channel.^20^ Note that the loading of the pores with water was full for all of the grain sizes, including the smallest size, and was controlled gravimetrically (see the Supporting Information).

**Figure 2:**
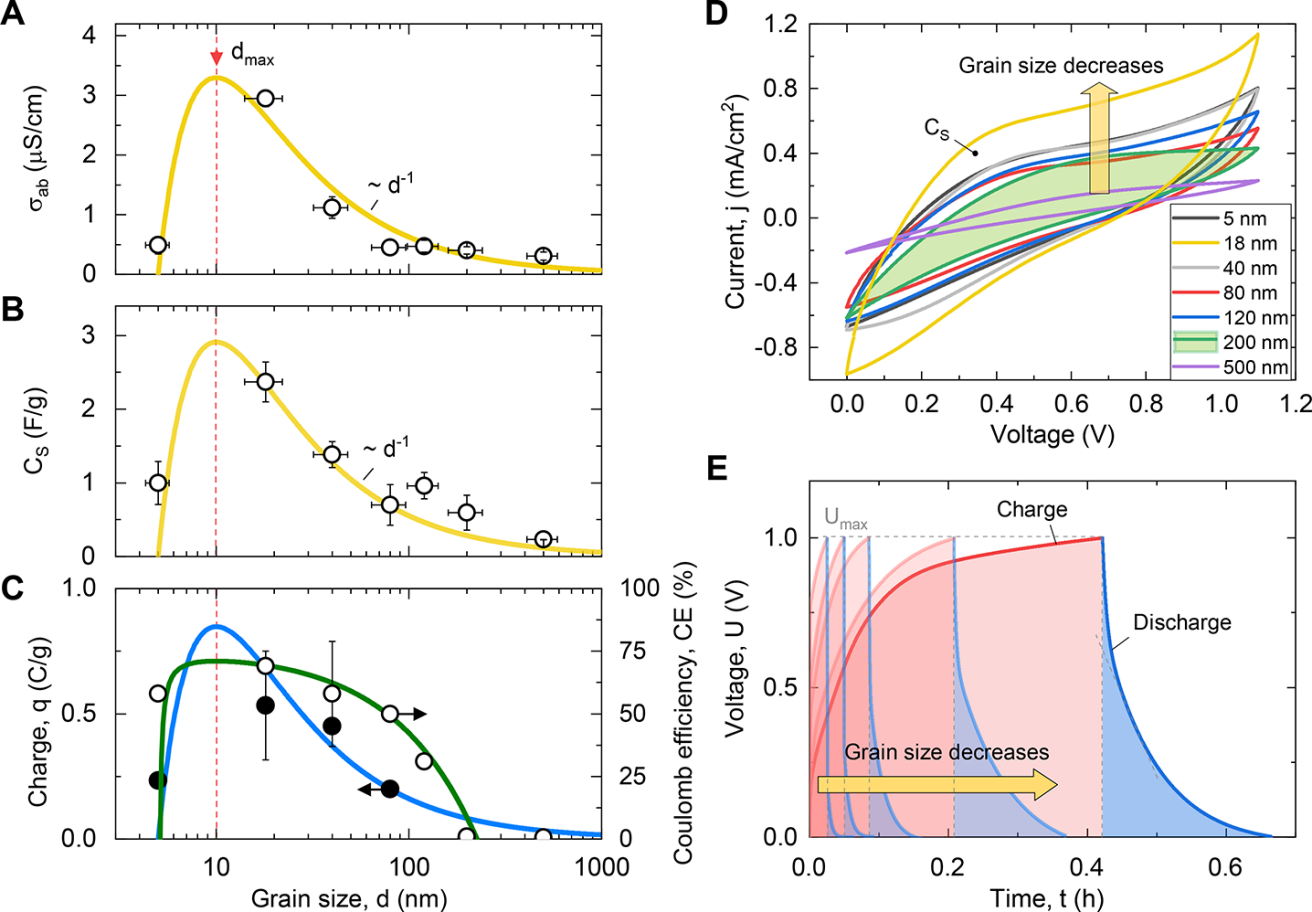
Electrodynamic characteristics of a sustainable confined-water accumulator. (A) Protonic direct current (DC) conductivity, σ_ab_, of the cell, measured between points a and b (Figure 1B). The dots are experiments, and the yellow lines are models according to the model discussed in the text. (B) Specific capacitance of the cell assembly calculated from the voltammograms. (C) Specific charge, *q*, and coulombic efficiency, CE, of the cell. (D) Cyclic voltammograms of the cell assembly for different grain sizes (see the legend). The area of the loops corresponds to the cell-specific capacitance *C*_s_ (see panel B). (E) Voltage evolution during a charge–discharge cycle at a current *j* = 0.4 mA for different grain sizes.

The cyclic voltammograms of the cells (Figure 2D) are also strongly dependent upon the grain size. The areas of the hysteresis loops are proportional to capacity *C*_s_ (Figure 2B). For large pores of fractions of micrometers, the loop opening is small as a result of the high resistivity of the separator. On the contrary, tiny pores have more significant hysteresis as a result of the cumulative effect of a larger specific surface and an increase in the proton conductivity of the separator. Note that the way we have filled the pores with water excludes the addition of impurities (see the Supporting Information). The conductivity changes as a result of the fractional increase of the interfacial water, which is affected by the surface.^18,37^ Thus, we had a pure size effect. The colored regions under the charge–discharge curves (Figure 2E) also show an increase in capacitance as *d* decreases. The shape of the charge–discharge curves is determined by resistance and diffusive charge redistribution.^38^ The electric charge is stored in the electrochemical double layer.^39^ The dominant charge carriers are excess protons and proton holes (H_3_O^+^ and OH^–^ ions). We cannot exclude hydrolysis reactions at the electrodes. However, the long-term tests (Figure S11 of the Supporting Information) showed stability of the cell capacitance. If H_2_ and O_2_ molecules form at the electrodes, the process is reversible and does not affect the cell properties.

The behavior reported above can be understood using the model of the simultaneous effect of bulk and interfacial water layers at the interconnected network of nanopores. We consider the inner pore space as the effective medium formed by an interfacial water layer of a constant thickness and the bulk water layer (panels F and I of Figure 1). When the pores are hundreds of nanometers (or larger), the ratio between *r* and *h* is such that the interfacial water layer of about 1 nm can be neglected. In this case, the pore electrodynamics is determined by the ordinary bulk water properties. In small nanopores of a few nanometers, *h* is about *r* and the interfacial water determines the cell electrodynamics. In a general case of intermediate pore sizes, the conductivity σ_ab_ and the capacitance *C*_s_ are determined by *F*(*r*) = *A* + *Bh*(*r* – *h*)*r*^–2^, where *F*(*r*) is either *C*_s_(*r*) or σ_ab_(*r*), *A* is either *C*_bulk_ or *R*_bulk_^–1^, and *B* is either *C*_IF_ or *R*_IF_^–1^ (Figure 1B and Figure S7 of the Supporting Information).

The parameter *h* in the equation above, which determines the thickness of the interfacial water layer, is the only unknown argument. Previous studies reported the thickness of the interfacial water layer *h* ≈ 1.5 nm.^10,11,13−16,18,39^ This value is consistent with the mean distance between the short-lived intrinsic ions of water.^26,40^ The best fit of the model to the experimental data with this value of *h* is shown in yellow in panels A and B of Figure 2. The parameters are given in Table S1 of the Supporting Information. Although there are no data points at the maxima of the curves, the simultaneous fit of σ_ab_, *C*_s_, CE, and *q* unambiguously gives *r*_max_ = 3 nm. This value is twice as large as *h*. Thus, the maximum conductivity and the capacitance are observed when the entire pore space is filled with interfacial water but the layers from the opposite walls do not yet overlap. In other words, any nanoconfined water can be treated as a superposition of bulk water and interfacial water, whose effective medium parameters depend upon their volume fractions. The size effect on the electrical conductivity of confined water is equivalent to increasing the frequency^32^ or adding electrolytes.^36,37^ However, high conductivity and capacity are achieved without foreign species. This fact positively distinguishes our device from those of other electric energy accumulators.

Figure 3 compares the characteristics of our device to those of other energy sources: accumulators, batteries, and fuel cells. Although our water-only accumulator is at an early prototype stage and hardly competes with commercial products, its power and energy densities are as high as 5 W/kg and 2.5 Wh/kg, respectively (see the red dot). These values are reasonable for a 1 mm thick membrane used in the model experiments with grain sizes. Reducing the separator thickness down to 0.5 and 0.3 mm showed an increase in power and energy density (see blue dots). Extrapolation to the technologically plausible separator thickness down to 10 μm, typical for commercial supercapacitors,^41^ yields the upper right corner of the green area (Figure 3). We expect that in future studies the application of two-dimensional (2D) materials, such as graphene, for the electrodes can additionally increase the surface energy density.

**Figure 3:**
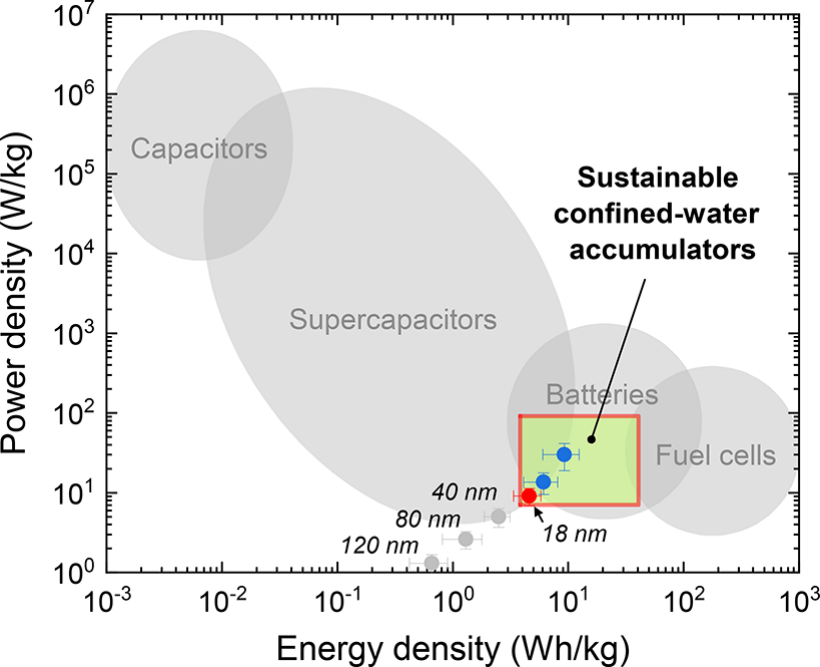
Electric energy sources by power and energy density. The dots show the experimental parameters obtained in this study for the cell with pure water as an electrolyte only. The gray points correspond to the cells with different pore sizes (see the numbers) of the thick model separator of 1 mm. The red point corresponds to the best parameters of the model cell with pore sizes close to the ideal. The blue points are for the same cell but with thinner separators of 0.5 and 0.3 mm. The green rectangle shows the area that the sustainable confined-water accumulators can occupy in the case of optimization suggested in this research. The boundaries of the gray areas are based on the latest experimental data.

The green area in Figure 3 overlaps with that of commercial batteries. Thus, our water-only nanofluidic device combines reasonable electric capacity with environmental neutrality. A new approach to the construction of aqueous energy systems discussed in this work provides an opportunity to supplement renewable energy generators with sustainable energy storage for stationary and portable applications throughout the complete device lifecycle from a raw material supply to recycling. Further studies on the interaction of water with different materials, especially natural materials, are needed to reach this goal.

In summary, we investigated the charge storage capability of confined water in nanopores of carbon-based electrochemical cell assemblies. We found that confined water is not the same as bulk water in terms of electrodynamics. The water-only battery prototype showed promise for environmentally neutral energy storage solutions. The optimal pore size for maximizing power and energy density was found to be 3 nm. Because water exhibits electrical double-layer formation near different surfaces,^5,21,22,40,42^ it is reasonable to expect that water will exhibit electrolyte-like behavior not only near carbon-based materials but in most classes of porous materials, such as sand and clays. We believe that our research will stimulate further exploration of water-only batteries to bring this nanofluidic electrochemical concept to application. The results of our study are also essential for a better understanding of the interfacial phenomena in biological transport and nanofluidic devices.

## Supporting Information Available

Sample preparation and treatment, measurement methods, Figures S1–S10, and Table S1 (PDF)
