# Supplementary material for: Confinement-Controlled Water Engenders Unusually High Electrochemical Capacitance
Source: J Phys Chem Lett. 2023 Jul 17;14(29):6572–6. doi: 10.1021/acs.jpclett.3c01498 (PMC10388349; doi:10.1021/acs.jpclett.3c01498)
Supplement: Supplementary file 1 — jz3c01498_si_001.pdf [file jz3c01498_si_001.pdf]

Supporting information

Confinement-controlled Water Engenders Unusually High Electrochemical Capacitance

Svetlana Melnik,[†] Alexander Ryzhov,[‡] Alexei Kiselev,[¶] Aleksandra Radenovic,[§] Tanja Weil,[∥] Keith J. Stevenson,[⊥] and Vasily G. Artemov*,[§]

†*Atmospheric Microphysics Department, Leibniz Institute for Tropospheric Research, Leipzig, 04318, Germany*

‡*Center for Low-Emission Transport, Austrian Institute of Technology, Vienna, 1210, Austria*

¶*Institute of Meteorology and Climate Research, Karlsruhe Institute of Technology, 76021 Karlsruhe, Germany*

§*Institute of Bioengineering, École Polytechnique Fédérale de Lausanne (EPFL), CH-1015 Lausanne, Switzerland*

∥*Max Planck Institute for Polymer Research, Ackermannweg 10, 55128 Mainz, Germany*

⊥*Skolkovo Institute of Science and Technology, 121205 Moscow, Russia*

E-mail: vasily.artemov@epfl.ch

This PDF file contains:

1. Methods
2. Samples preparation
3. Samples cleaning
4. Surface area determination

5. Filling cells with water
6. Electrochemical measurements

- Figures S1 – S10
- Table S1

1. Methods

To study the charge storage capability of the water-only cell, we used dielectric spectroscopy, cyclic voltammetry, and galvanostatic techniques. In short, by applying an alternating electric field, we tested the electrical impedance and obtained the electrical conductivity and the dielectric constant, which are related to the mobility of the protons and the polarisability of the water-solid interface. The electrochemical measurements were carried out using a commercial potentiostat (see Sec. 6 below). To ensure the purity of water in the pores, they were filled by capillary condensation in an atmosphere of saturated water vapour. The surfaces of the carbon and diamond nanoparticles were cleaned by centrifugation of nanopowders in distilled water several times, followed by drying at a high temperature. The integrity of the cell was achieved by mixing up to 5% by weight PTFE into the initial powder (main text Fig. 1D). Note that due to the dense packing, the fraction of water confined to the pores was approximately the same for all fabricated cells regardless of grain size d, while the size of the percolating nanovolumes of water varied.

2. Samples preparation

Nanodiamonds powders with narrow grain size distributions around 5, 18, 40, 80, 120, 200, and 500 nm were purchased from Adámas Nanotechnologies (Fig. S1). Carbon black powder with grain-size distribution around 40 nm was purchased from Sigma-Aldrich. The powders were carefully cleaned before use (see next section). Electrochemical heterostructures (cells) were pressed from the powders in a stainless-steel press form and consisted of 3 layers (electrode-membrane-electrode) approximately 1 mm thick each and 5 mm in diameter. We

used a standard press form and a pressure of up to 20 tons per cm^2. Figure S2 shows the structure of the cell and layers at different scales. To increase the mechanical stability of cells, carbon particles were mixed with 5% wt. of polytetrafluoroethylene (PTFE) or polyethylene glycol (PEG). Note that no difference was found between these 'glue' materials, indicating a lack of effect on confined water dielectric properties. A minimum of 15 cells of each grain size (about 100 cells in total) were fabricated to verify reproducibility and minimize the error bars of data points. Cracks (Fig. S2 C, D), and short-circuit between the electrodes, were observed for some samples, leading to a decrease in cells performance or a partial loss of percolation, or enlarged effective resistance of the cell. These samples were excluded from the data analysis.

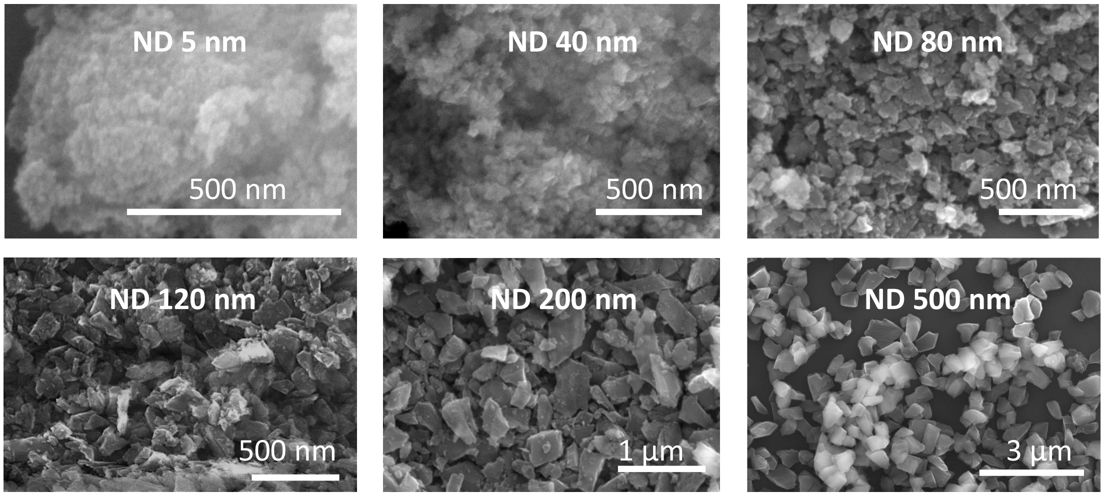

Figure S1: **Scanning Electron Microscope (SEM) images of nanodiamond (ND) powders of different grain sizes.**

3. Samples cleaning

The powders of nanodiamonds and carbon black were preliminary cleaned multiple times to avoid the influence of surface contaminants on the dielectric properties of water confined to the matrix of the pores formed by the grains of the host materials. To do this, the obtained from the manufacturer powders were dissolved in deionized water. We used Milli-Q Direct

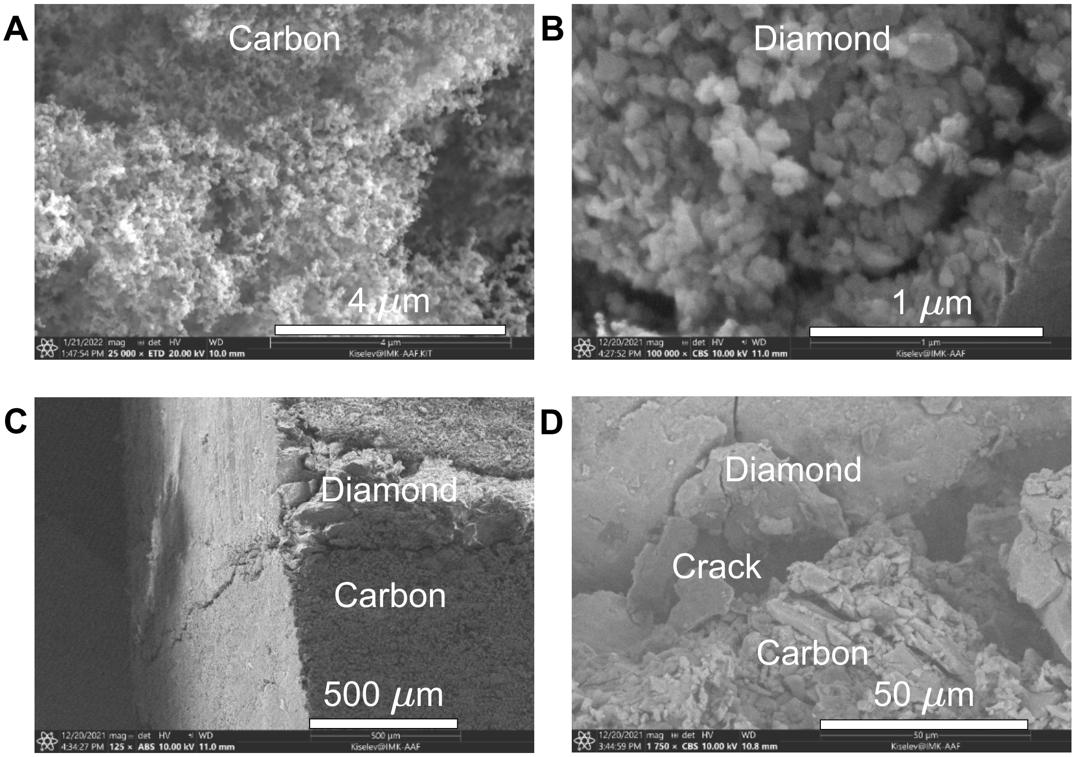

Figure S2: **Scanning Electron Microscope (SEM) images of the cell.** (**A**) Carbon electrodes. (**B**) Diamond membrane (80 nm). (**C**, **D**) Different cross-sections of the cell assembly.

Water Purification System and 18 MOhm·cm water. Then the mixture was centrifuged to separate the water and the powders, and the pH and DC conductivity of the brine were measured. The procedure was repeated many times until a complete stabilization of the measurements. Typically, about 10 cycles were needed to obtain no further change of conductivity and pH value within the accuracy of 0.05 units. After the washing, the samples were annealed in a laboratory oven at 200°C. The analysis of washed powders consisted of elemental analysis, infrared spectroscopy, and titration previously described elsewhere.[1] These methods revealed atoms of oxygen, silicon, and chloride on the surface of our carbon-based materials, but their concentration was less than 0.01% of the concentration of native carbon atoms. Moreover, the normalized-on-the-surface-area concentration of the IR-active surface groups was shown to decrease with the decrease of the grain size d, while the ionic (protonic) conductivity of water between the grains of these materials was shown to increase instead. Thus, the dielectric properties of cells were attributed to the confinement-changed properties of water, but not to the surface chemistry.

4. Surface area determination

The Quantachrome Autosorb instrument was used to determine the specific surface area (SSA) of the samples. The argon gas adsorption/desorption isotherms were measured at 87.13 K (Fig. S3). The SSA was calculated using standard Brunauer–Emmett–Teller (BET) analysis and was measured for both the powders and the compressed ceramics (pellets or membranes) as a function of the grain size d (Fig. S4). An inverse proportionality of the SSA (see the blue line) on d corresponds to an increase of the surface area of the samples proportionally to the rise of the surface area of the grains following the decrease of their diameter d. A good coincidence of SSA of powders and pellets was observed. Thus, the porosity of the pellets was open, and all the pores were available for the molecular species.

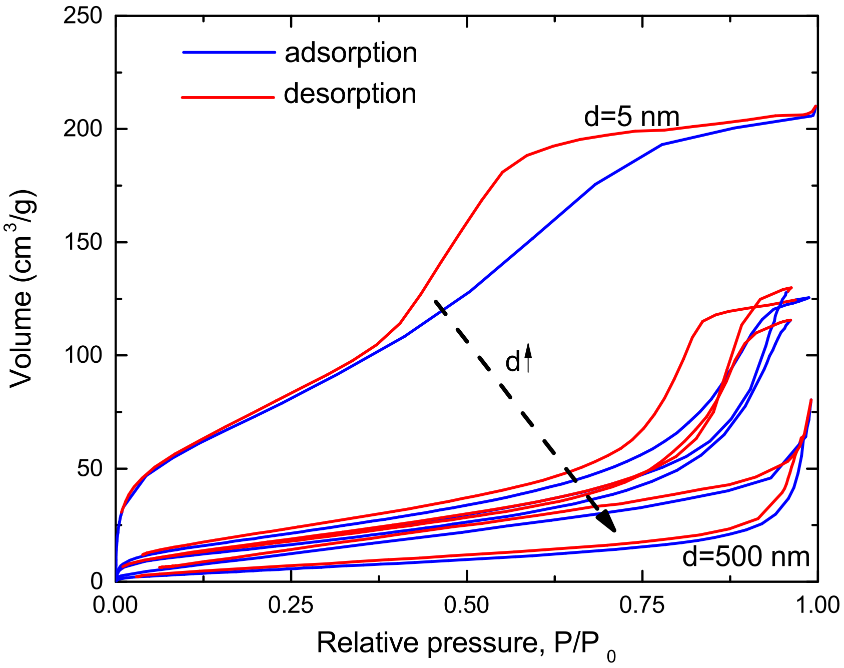

Figure S3: **Adsorption/desorption isotherms of argon on nanodiamond membranes.** Samples exhibit mesoporosity as indicated by the adsorption/desorption hysteresis. A sharp increase in the volume of adsorbed gas at $P/P_0 > 0.9$ indicates a capillary condensation.

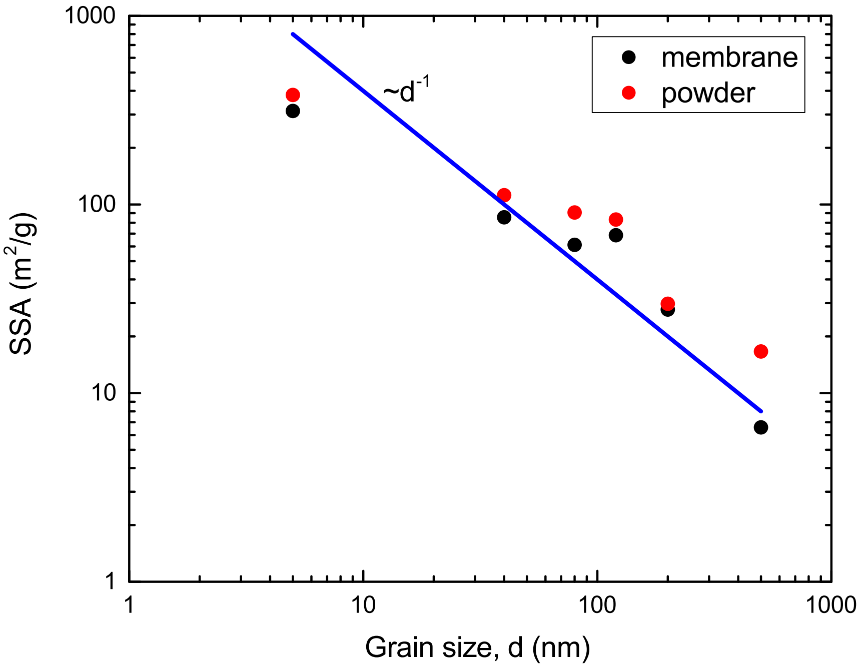

Figure S4: **Specific surface area (SSA) of nanodiamonds.** The red dots are for powder, and the black dots are for pellets made of powder. The SSA was calculated from Ar adsorption isotherms at 87.13 K (see Fig. S3). The blue line corresponds to the surface-to-volume ratio (d^{-1}) of the grains typical for spherical particles.

5. Filling cells with water

The porosity of our electrochemical cells was obtained by the comparison of the volume, mass, and density of the raw materials and the pressed heterostructures. It was between 35 and 40%, which is close to the closely packed limit. The free space between the grains was filled with water and was controlled by gravimetric analysis. The following procedure was developed to assure a complete pore filling. The cells were placed in a desiccator and pumped within 30 minutes to remove the air from the pores. Then, samples were stored in the same desiccator in the atmosphere of the saturated water vapor for 10 hours. The mass gain was controlled gravimetrically and showed a complete occupation of the pores by water. The total mass of the device (carbon electrodes, separator, and water) was used for the calculation of the specific parameter shown in Fig. 3 of the main text. The closed Teflon casing with inserted parallel-plate gold electrodes was used to prevent cell drying during the measurement. The cells' mass was controlled before and after the measurements and showed no decrease within several hours.

6. Electrochemical measurements

The complex impedance (EIS) of the cells was measured using a Keysight E4980A analyzer operating in the frequency range from 20 Hz to 2 MHz. The parallel-plate-capacitor geometry with circular polished electrodes covered with gold was used. The electrodes' diameter was 5 mm, and the thickness of the cell was approximately 3 mm. All the measurements were performed at 22±2 °C. Figure S5 shows the typical EIS data. The DC (see the low panel and low frequencies around 10 Hz) electric conductivity of the dry cell was approximately five orders of magnitude lower than that for wet samples. The electric conductivity of pure water is shown in gray for comparison and corresponds to $5.5 \cdot 10^{-6}$ S/m. The dielectric constant (see the upper panel and low frequencies), on the contrary, was up to five orders of magnitude than that of bulk water and up to seven orders of magnitude larger than that of dry sample (out of the graph, approximately 3 units of the dielectric constant). This

additional polarization of wet samples is due to the diamond/water interface, presumably due to the formation of an electrochemical double-layer of intrinsic ionic species of water.

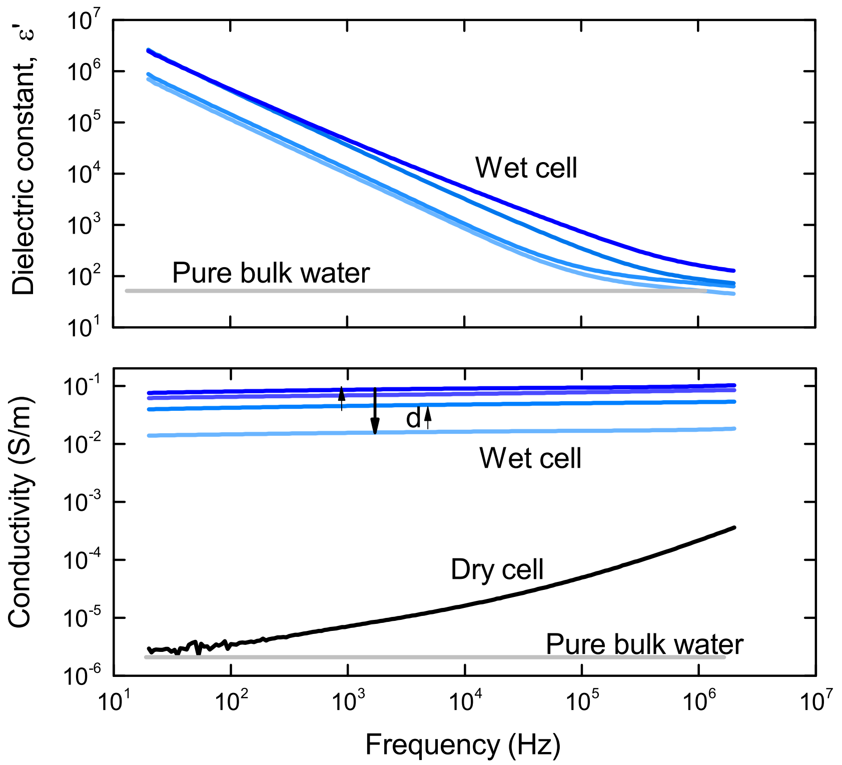

Figure S5: **Dielectric spectra of the cells in the frequency range from 20 Hz to 2 MHz.** The upper panel is for the real part of the dielectric functions, and the bottom panel is for the dynamic conductivity. The shades of blue correspond to the water-filled porous cells with different grain sizes d. The black curve is for the dry cell. The gray horizontal lines are for the dielectric constant and the dynamic conductivity of pure bulk water.

A BioLogic impedance analyzer was used for Cyclic Voltammetry (CV) measurements. Two-electrode configuration was applied to the same samples used in EIS experiments. The current density of the galvanostatic test was ramped from 1 to 20 mA/cm^2. The cell voltage was always maintained below 1 V to avoid massive hydrolysis. Each sample was subjected to several 0-1-0 V cycles with a scanning rate of 5 mV/s (see Fig. 2d of the main text). Voltammograms were used to calculate the capacitance, which is proportional to the hysteresis area. The latter was normalized by the voltage window. To obtain specific capacitance, the values were divided by the cell mass (Fig. 2c of the main text), i.e., Teflon casing and

metal electrodes were excluded.

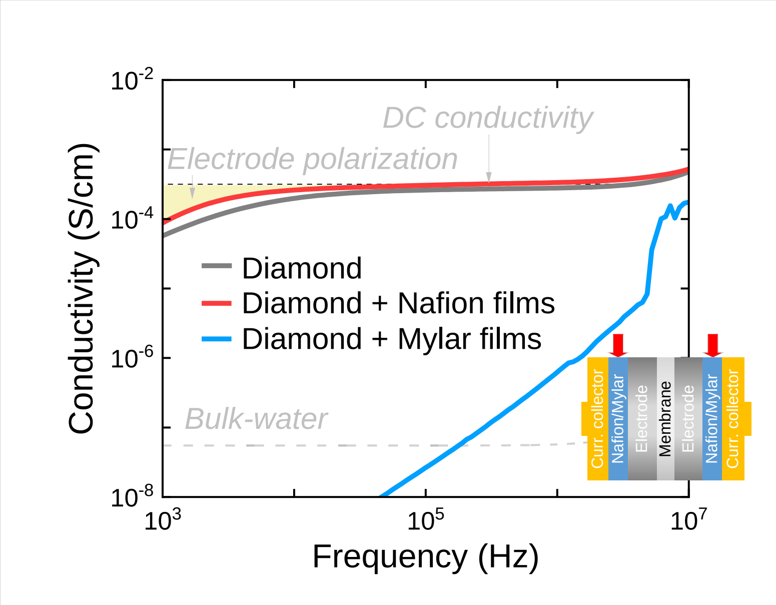

Figure S6: **Electrical conductivity spectra** (1 kHz to 10 MHz) of cells without blocking electrodes (grey), with Nafion (red), and with Mylar (blue) films placed between the cell and the electrodes (see inset). The conductivity of pure bulk water is shown by a dashed line for comparison. The inset scheme shows the layout of the experimental cell. Red arrows indicate the position of additional layers, which were placed to confirm the protonic conductivity.

Table S1: **Fit parameters of the model to the experimental data points in Fig.2.(see main text)**. A is either C_{bulk} or R_{bulk}^{-1}, and B is either C_{IF} or R_{IF}^{-1} (see Fig. 1B of the main text)

	Counductivity (S/cm)	Capacitance (F/g)
A	$5.5{\cdot}10^{-8}$	$3.4{\cdot}10^{-11}$
B	$1.5{\cdot}10^{-2}$	11.5

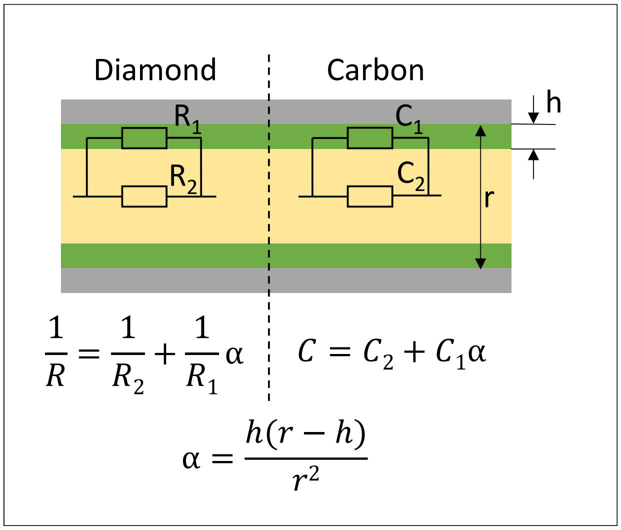

Figure S7: **On the derivation of the model for conductivity and capacitance** (see yellow lines in Fig.2, A and B, of the main text and the corresponding formula). Gray layers represent the walls of the diamond (left) and the carbon (right). Green and yellow layers are interfacial and bulk water, respectively. Formulas are for the equivalent resistivity (R) and capacitance (C), represented by the equivalent schemes. The coefficient α is a geometrical factor reflecting the pore's relative amount of bulk and interfacial water for a general case of the random pore shape.

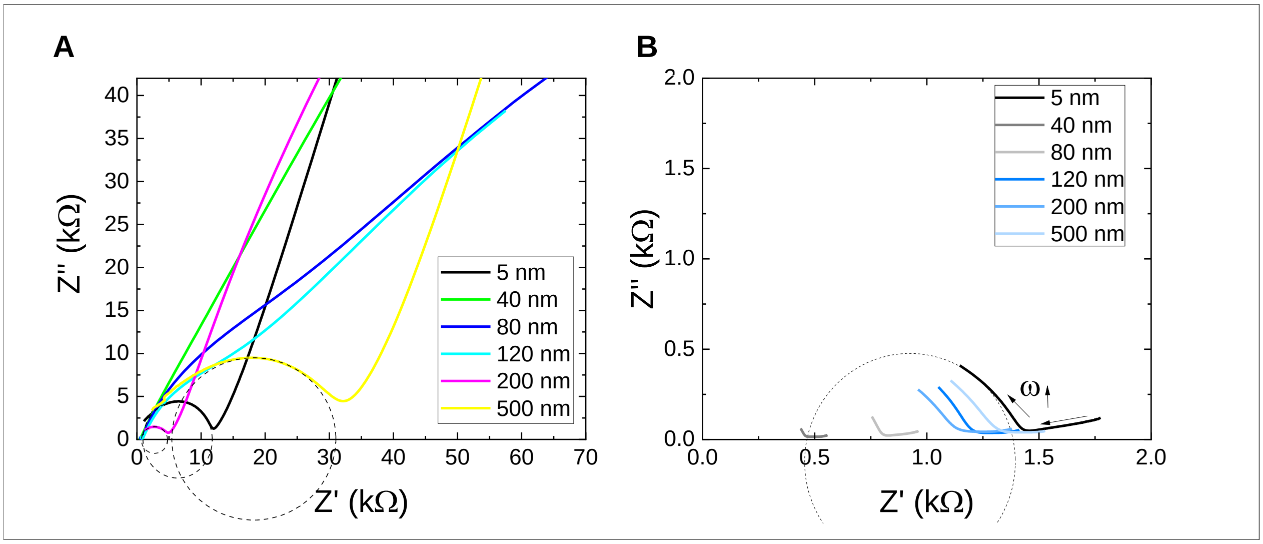

Figure S8: **Nyquist plots.** Complex impedance of the nanodiamond separator (A) and the carbon-diamond-carbon cell assembly (B). Different colors correspond to the different grain sizes of diamonds (see legend).

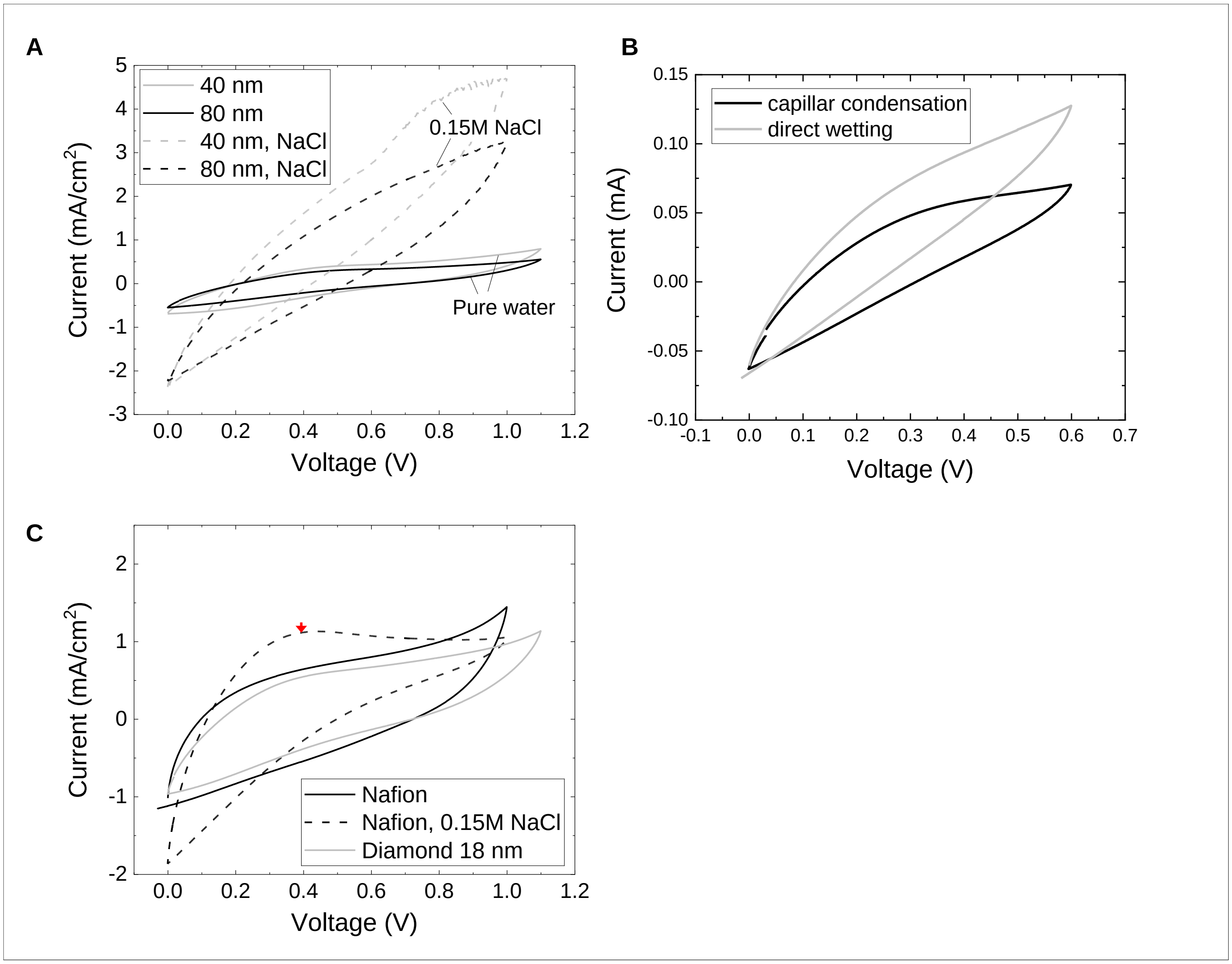

Figure S9: **Cyclic voltammograms.** (A) Cell with a nano-diamond membrane filled with pure water (solid) and with a 0.15 M solution of sodium chloride (dashed). The gray and black curves correspond to 40 and 80 nm grain sizes. (B) Cell with a nano-diamond membrane filled by capillary condensation (black line) and direct wetting with tap water (gray). (C) Cell with a 1 mm nano-diamond membrane filled with pure water (gray), for supercapacitor with a 250-micron Nafion membrane filled with pure water (black solid), and with 0.15 M solution of NaCl (black dashed). The red arrow shows the part caused either by the stuck of the nonprotonic ionic species at the Nafion surface or by the parasitic redox reaction on the electrodes involving Na^+ and Cl^- ions.

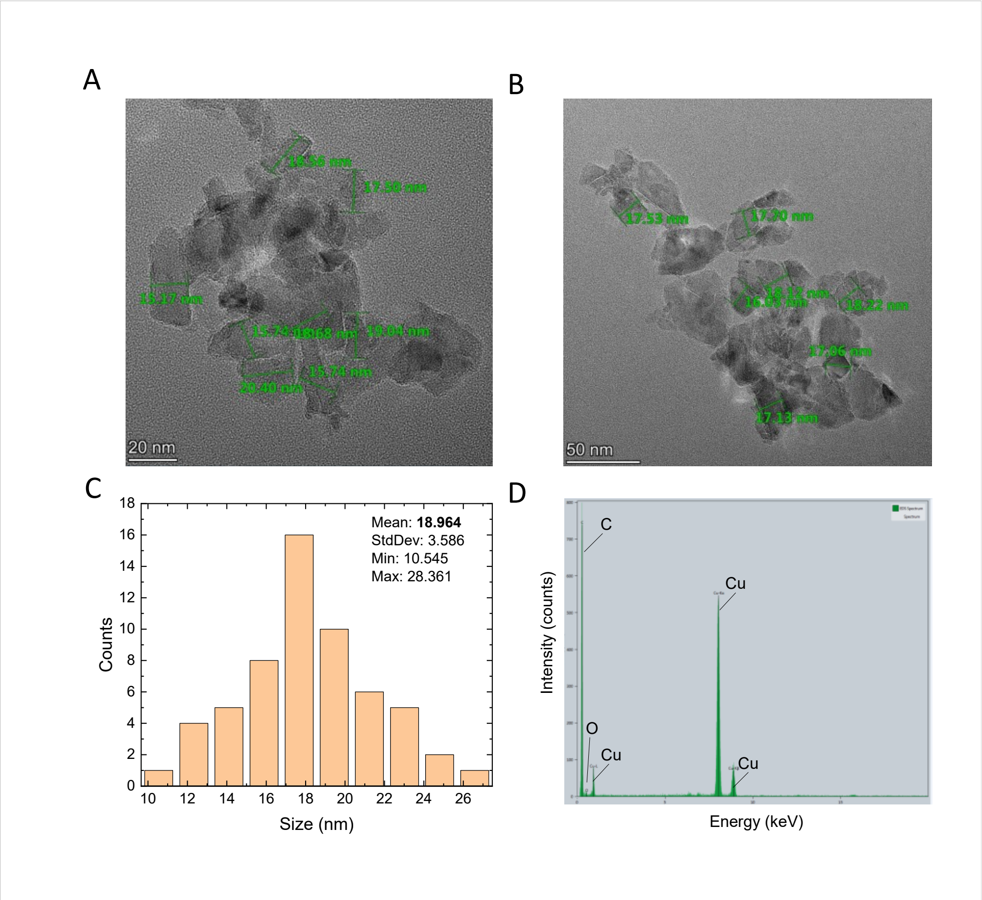

Figure S10: **Characterization of nanodiamond powder**. (A and B) TEM pictures of 18-nm grains, (C) grain size distribution, and (D) EDS spectrum. Peaks of Cu on the spectrum correspond to the used substrate.

References

(1) Artemov, V.; Uykur, E.; Kapralov, P.; Kiselev, A.; Stevenson, K.; Ouerdane, H.; Dressel, M. Anomalously high proton conduction of interfacial water. *J. Phys. Chem. Lett.* **2020**, *11*, 3623–3628.
